# Femoral Neck Fractures in HIV-Positive Patients: Analysis of 10 Years Short-Term Post-operative Complications

**DOI:** 10.5704/MOJ.2111.010

**Published:** 2021-11

**Authors:** A Manzotti, MM Larghi, A Schianchi, M Grassi, C Pullen, P Cerveri

**Affiliations:** 1 Department of Orthopaedic and Trauma, Luigi Sacco University Hospital, Milan, Italy; 2 Department of Orthopaedics, University of Milan, Milan, Italy; 3 Department of Orthopaedics, The Royal Melbourne Hospital, Victoria, Australia; 4 Department of Bioengineering, Politecnico di Milano, Milan, Italy

**Keywords:** HIV, femoral fracture, surgery complications

## Abstract

**Introduction:**

Aging and effect of antiretroviral therapy on bone mass could increase the risk of femoral neck fractures (FNF) in HIV patient. The aim of this study was specifically to determine whether intracapsular FNF in HIV-positive patients are more prone to short-term post-operative complications than similar fractures occurring in HIV-negative patients.

**Materials and methods:**

A group of 25 HIV-positive patients with intracapsular FNF were enrolled and matched to HIV-negative patient with similar fractures according to gender, age, a modified Charlson Comorbidity Index (CCI), fracture classification, surgical treatment and time interval between fracture event and surgery. For each group, length of stay, surgical time, early clinical outcomes and short-term surgical and medical complications were compared to determine the impact on the early outcome.

**Results:**

At the time of the fracture occurrence, 56% of HIV-positive patients were on antiretroviral therapy and 12% started with therapy in the perioperative period. At three months follow-up, there were no statistically significant differences between the two study groups in length of stay, Harris hip score and total number of early complications. However, a statistically significant increase in urinary tract infections and longer surgical time using hip sliding screw fixation were seen in the HIV-positive group. The poorest post-operative result was seen in a patient who failed to adequately adhere to the HIV therapy protocol.

**Conclusions:**

This study failed to show any statistically significant increase in short-term complications or worse clinical outcomes for intracapsular FNF in HIV-positive patients compared to HIV-negative patients to recommend their treatment in dedicated centres.

## Introduction

Femoral neck fractures (FNF) are an increasing problem. An improvement in living standards and the greater effectiveness of treatments for chronic diseases has resulted in an older mean age in world population with a well-documented consequent higher incidence of femoral neck fractures^1^. Likewise, life expectancy in HIV-positive patients has also increased significantly, due to successful medical management with Highly Active Antiretroviral Therapy (HAART)^2^. Whilst HAART therapy is effective in treating HIV infection it also plays an important role in suppressing osteoclastogenesis, inhibiting osteoblast differentiation and causing a significant bone mass index (BMI) reduction^3,4^. In addition, HIV infection is more common in patients with methadone/opioid addiction and Hepatitis C virus (HCV), both conditions adversely affecting bone quality^5^.

Many clinical studies have been published documenting the clinical outcomes of orthopaedic surgeries in HIV-positive patients often with controversial results. Some authors suggest that HIV-positive patients have higher rates of wound infection and non-union after internal fixation than HIV-negative patients^6,7^. Other studies have suggested that HIV infection does not correlate with a higher rate of post-operative infection and fracture non-union^8-10^.

The purpose of this study was to determine specifically whether intracapsular femoral neck fractures in HIV-positive patients are more prone to short-term post-operative complications than similar fractures occurring in HIV-negative patients and consequently best addressed in a multi-specialist hospital with a dedicated infectious diseases unit.

## Materials and Methods

From 2007 to 2017, 1409 proximal femoral fractures that presented to a single, urban, University-affiliated Hospital were identified using an electronic search based on the International Classification of Diseases and Current Procedural Terminology (CPT) codes (ICD 820.00, ICD 820.01, ICD 820.02, ICD 820.03, ICD 820.09, ICD 820.11, ICD 820.12, ICD 820.20, ICD 820.21, ICD 820.22). The hospital was equipped with both a referral centre for infectious diseases and a Level II Emergency Department. Of the 1409 proximal femoral fractures, 50 intracapsular femoral neck fractures (3.7%) occurred in HIV-positive patients and 25 of these patients were enrolled in the study (Group A). The exclusion criteria for the study patients were: missing data regarding both the immunological status and any clinical/surgical data in the first three months of post-operative follow-up. Each HIV-positive patient was subsequently matched to a HIV-negative patient with a similar femoral neck fracture (Group B) treated surgically in the our Institution. Matching-criteria were: gender, age (+/-6 years), modified Charlson Comorbidity Index (CCI) (+/-5 points), femoral fracture type according the AO classification considering intracapsular femoral neck fracture type A or B, surgical procedure following our department guidelines and time interval between fracture occurrence and surgery (cut-out 48 hours). The standard guidelines adopted in our department for all femoral neck fractures (including in HIV-positive patients) advocate sliding hip screw for A1 type fracture, pertrochanteric nails in all A type fracture, cannulated screw for undisplaced B1 and B2 fracture in patients below 65 years, hemiarthroplasty for type B displaced fracture in patients over 70 years and total hip arthroplasty in type B displaced fracture in patients below 70 years or pre-existing hip arthritis. All the fractures included in the study were surgically managed by orthopaedic consultants belonging to the same surgical team (nine orthopaedic consultants all with a similar surgical expertise and all on-call for the orthopaedic trauma department). The Charlson Comorbidity Index (CCI) classifies co-morbid conditions predicting mortality risk with a score ranging from 1 to 6 for 19 pathological conditions (including HIV-positivity). The CCI is an easily applicable method for the pre-operative assessment of comorbidities even in patients undergoing orthopaedic surgery^11^. In our matching process, the HIV-positivity score (max 6 points) in CCI index was not included to achieve a homogeneous distribution of comorbidities between the two groups. In HIV-positive group, patients were assessed for: viral load, CD4 cells count and the use of HAART within three months prior to surgery. A viral load less than 50 copies/ml was considered undetectable and CD4 count lower than 200 cells/l as a severe immunodeficiency^12^. In both groups, length of stay, percentage of patients operated on within 48 hours of fracture, surgical time, 3-month post-operative Harris Hip Score and presence of short-term complications was assessed. These data have been routinely collected during the ordinary three months rehabilitation follow-up and orthopaedic visit, as institute protocol. Short-term complications (within three months of index surgery) were divided into surgical and medical groups according to Carpintero *et al*^13^. We considered post-operative anaemia, defined as patients requiring a post-operative blood transfusion, and urinary tract infections (UTIs), defined as urine culture colony counts >100000 colony-forming units per millilitre (CFU/mL), as medical complications. Haemoglobin value <8g/dl was used as a decision threshold in all patients undergoing red blood cell transfusion. Surgical complications included post-operative mortality within three months of index surgery, any revision surgery, hip pain (Numeric Rating Scale value: ranging from 0 to 10), limping (positive Trendelenburg sign) and limb length discrepancy (more than 1.5cm). All surgical wound problems including aseptic dehiscence, hypoesthesia and superficial or deep infections were also considered surgical complications.

Statistical analysis was performed using the Odds Ratio with a 95% confidence interval for systemic and local post-operative complications immediately after surgery and at three months. Statistical analyses were performed using MedCalc for Windows, version 15.0 [MedCalc Software, Ostend, Belgium]. The paired t test and chi-squared test were performed for comparisons and the significance level was taken as P<0.05 for all values.

## Results

FNF in the HIV-positive group occurred in a younger population (mean 57 years) compared to the control group (mean 63 years); however this value did not have a statistical significance (P-Value=0.13). In both groups, there were 17 (68%) B-type proximal femoral fractures and 8 (32%) A-type fractures according to AO classification and 20 (82%) patients in both the groups were treated within 48 hours of the fracture occurrence (Table I).

**Table I:** Demographic data

Demographic Data	HIV+ (Group A) Mean +/- STD	HIV- (Group B) Mean +/- STD
Age	57.4 (+12.8)	63 (+13)
Gender	60% M	60% M
Modified CCI Score	1.9 (+1.6)	1.9 (+1.6)
CD4+ count	536 (+246)	/
Viremia	72% <37 NR	/
Surgery performed within 48 hours	82%	82%
Length of stay (days)	11.6 (+8.8)	10.31 (+6.12)

In group A, the mean CD4+ count at the time of surgery was 536 (STD +246) with an undetectable viremia in 18 (72%) patients (mean<37 copies/ml) and 14 (56%) patients, despite different antiretroviral protocols, were already on HAART pre-operatively while three (12%) started HIV antiretroviral therapy in the perioperative period. Only two cases (8%) of high-energy trauma were seen in the HIV-positive group compared to eight (32%) cases in the control group, showing a statistical significant difference (P-Value = 0.035).

The mean length of stay was 11.6 days (STD +8.8) for patients in Group A and 10.30 days (STD +6.12) for those in Group B. In both the groups all hemiarthroplasty and total hip arthroplasty procedures were performed using an anterolateral approach. The mean surgical time (minutes) in Group A was 96 (STD +31) for hemiarthroplasty, 112 (STD +20) for total hip arthroplasty, 90 (STD +8) for fixation with a sliding plate screw, 44 (STD +23) for fixation with cannulated screws and 47 (STD + 20) for peritrochanteric nail. In Group B, the mean surgical times (minutes) were: 100 (STD +29) for hemiarthroplasty, 110 (STD +23) for total hip arthroplasty, 70 (STD +7) for sliding hip screw, 45 (STD +15) for fixation with cannulated screws and 41 (STD + 23) for peritrochanteric nail. There were no statistically significant differences in surgical time for the different surgical procedures except for hip sliding screw procedure with a significant longer surgical time in Group A (p<0.01) (Table II).

**Table II:** Three months follow-up Harris Hip Score and surgical time

	HIV+ (Group A) Value +/- STD	Harris Hip Score HIV- (Group B) Value +/- STD	P-Value
Harris Hip Score	74.52 +/-17.51	78.62 +/-13.33	0.36
	**HIV+ (Group A) Mean +/- STD**	**Surgical Time (minutes) HIV- (Group B) Mean +/- STD**	**P-Value**
Hemiarthroplasty Total Hip Arthroplasty	96 min (+/-32”) 112min (+/-20)	100mim (+/-29’) 110min (+/-23”)	0.85 0.89
Sliding Hip Screw Cannulated Screws	90min (+/-8’) 44min (+/-23’)	70min (+/-3’) 45min’ (+/-15)	<0.01 0.94
Pertrochanteric Nails	48min (+/-20)	41min (+/-23)	0.71

The rate of surgical complications was the same in the two groups with no statistical differences (Table III). Mortality within three post-operative months occurred in three patients; of these two patients were HIV-negative and one HIV-positive. In each case the cause was secondary to pre-existing co-morbidities unrelated to the femoral fracture (p=0.55). Three patients (12%) in Group A and one patient (4%) in Group B underwent revision surgery because of implant related problems (p=0.30).

**Table III:** Surgical and medical complication observed

	HIV+ (Group A) % (n)	Surgical complications HIV- (Group B) % (n)	ODD RATIO	IC 95%	P-Value
Mortality	4% (1)	8% (2)	0.47	0.04-5.65	0.56
Re-intervention	12% (3)	4% (1)	3.27	0.32-33.84	0.31
Pain (NRS>5) at 3 months	20% (5)	24% (6)	0.79	0.21-3.03	0.73
Positive Trendelenburg Sign	16% (4)	8% (2)	2.19	0.36-13.22	0.39
Leg length discrepancy	12% (3)	16% (4)	0.72	0.14-3.59	0.69
in surgical site infection	12% (3)	0% (0)	7.93	0.39-162.07	0.08
Other wound problems	0	8% (2)	0.18	0.01-4.04	0.16
	**HIV+ (Group A) % (n)**	**Medical complications** **HIV- (Group B) % (n)**	**ODD RATIO**	**IC 95%**	**P-Value**
Post-operative anaemia	48% (12)	24% (6)	2.92	0.87-9.78	0.08
Urinary tract Infection	28% (7)	4% (1)	9.33	1.05-82.7	0.04
	**HIV+ (Group A) % (n)**	**Total rate of complications** **HIV- (Group B) % (n)**	**ODD RATIO**	**IC 95%**	**P-Value**
HIV+ (Group A) % (n)	HIV- (Group B) % (n)	ODD RATIO	IC 95%	P-Value	

At three months post-operative according to Carpintero classification there were no statistical significant differences in post-operative complication except for both superficial surgical site infections and UTIs.

Five (20%) of the HIV-positive patients reported hip pain (mean NRS value>5), compared to 6 (24%) of the HIV-negative patients (p=0.73). A positive Trendelenburg sign was present in four (16%) of the HIV positive patients and in two (8%) HIV-negative patients (p=0.38). A post-operative leg length discrepancy greater than 1.5cm was observed in three (12%) of the HIV-positive group and in four (16%) of the HIV negative group (p=0.68). Superficial surgical site infection (all with positive cultures) occurred in three (12%) HIV-positive patients (two treated with sliding hip screws, one with hemiarthroplasty) in and none in HIV-negative patients, with no statistical significant difference (p=0.07). In Group B, one patient had an aseptic wound dehiscence because of poor suture reabsorption and another patient reported post-operative peripheral hypoesthesia close to the surgical (p=0.31). The mean Harris Hip Score at three months post-operative in the HIV-positive patients and control group was 74.52 (STD +/- 17.51) and 78.62 (STD +/- 13.33), respectively and the difference was not statistically significant (p=0.16) (Table II). No statistical difference (p=0.08) was found in the occurrence of post-operative anaemia despite a higher number of patients requiring blood transfusion in Group A (12) compared to Group B (six). There was a significant increase in the number of HIV-positive patients developing post-operative urinary tract infection (seven, 28%) compared to HIV-negative patients (one, 4%) (p=0.021) (Table III). We consequently assessed the immunological status of these seven HIV-positive patients who had developed urinary tract infection. One patient was HIV treatment naive with a viremia >20000 and a CD4 + count of less than 200/mcl, four patients had a T-Helper lymphocyte count <350/mcl with an undetectable viral load and the one had a CD4 + <500/mcl and a viral load >50.

## Discussion

In literature there are few studies examining the early outcomes of fracture fixation in HIV-positive patients in developed countries. The published literature is of only one study specifically dealing with surgery on the femur in HIV-positive patients^14^. The authors found a low rate of surgical site infection, and no immune status related complications. They advocated undertaking emergency procedures for HIV-positive patients only in a tertiary facility with infectious disease expertise. However, this report included only one acute femoral fracture in a relatively small sample including osteonecrosis, implant failures and osteomyelitis.

In our study the majority of FNF in HIV-positive patients occurred in males by a ratio of Male/Female: 1.5 in accordance with the literature but in contrast with the reports on the general population where the majority of these fractures occur in females (mean ratio Male/Female = 1:2)^15,16^.

HIV-positive patients quite often present with a deficient nutritional status and Hepatitis C virus or Hepatitis B virus coinfection, which might have a negative influence on bone metabolism^4,5,17^. Furthermore higher serum values of Tumour Necrosis Factor-alpha (TNF-α) and Osteoprotogerin (OPG) in HIV-positive patients are thought to promote bone resorption^3-7^. Similarly in the literature several authors have demonstrated earlier osteopenia development in HIV-positive patients secondary to a potential chronic inflammation status and the side effects of antiretroviral therapies on bone metabolism^17^. In our study, despite no statistical significance between the two groups in term of age, FNF occurred in a younger population (mean 57 years) compared to the control group (mean 63 years) in accordance with the epidemiological data on fractures occurrence in HIV-positive patients reported in the literature^18,19^. Likewise we observed a higher statistical significant incidence of low energy trauma in HIV-positive population and we hypothesise a poorer bone quality as a possible explanation for these two findings^5^. In our study 56% of the patients in HIV-positive group were already on HAART and further 12% started HIV antiretroviral therapy during the perioperative period. However, because both a not-homogeneous antiretroviral therapy protocol in term of doses, length of assumptions, and the relative small sample size, we could not perform any statistical analysis for significant correlation among HAART therapy, trauma mechanism and fracture occurrence.

In accordance with literature^8-10^, we did not detect any statistically significant difference in the total rate of both surgical and medical complications between HIV-positive and the HIV-negative patients. In the HIV-positive group, one patient underwent two revision surgeries because of implant breakage and a subsequent infection at the surgical site. This patient was poorly adherent to the anti-viral therapy, with both the CD4 count and viremia not adequately controlled. In another two cases requiring revision surgery, one secondary to high energy trauma and the other implant intolerance, we found no reasonable correlation between the re-interventions and HIV status.

We noted a significantly longer duration surgical procedure for Group A patients specifically when fixation was undertaken using sliding hip screws. Even if several variables such as fracture configuration and surgeon expertise can occur in influencing surgery time, in our study all the orthopaedic consultants involved in surgery had a similar surgical expertise following a standardised protocol in term of surgical technique indications. This ensured greater caution by the surgeons to avoid intra-operative exposure to HIV infection in a relatively wider and bloodier surgical exposure.

We observed a significantly higher incidence of post-operative urinary tract infections in HIV-positive patients and hypothesise that this may be due to either a state of immune deficiency or a lack of control of the underlying pathologies^20-24^. We specifically assessed the immunological status of these seven HIV-positive patients with a urinary tract infection and we could not detect any correlation between patient immunological status and higher rates of urinary tract infection.

In the present study we registered a 12% superficial surgical site infection rate in HIV-positive group compared to none in the control group and despite a statistical no significant difference (P-Value=0.07) this could show in Group A trend towards a higher surgical site infection risk even considering significant longer surgical time in selected surgical procedures^22-24^. One patient with surgical site infection was in HAART at surgery times and two patients were started HAART during recovery. Ma *et al*^24^ stated that analyses of CD4, ESR, and PCT could help in predicting the incidence of surgical site infection in HIV-positive patients but according to our results it still remains to be proven whether poor adherence to anti-viral therapy is one of the modifiable risk factors for surgical site infection in HIV-positive patients undergoing surgery for femoral fractures . In our analysis, and in accordance to Baburam *et al*^23^, no statistically significant difference at three months was seen in post-operative pain, Harris hip scores, limb length discrepancy and wound healing between the two groups and even the surgical site infection observed in the positive group did not cause any adverse effects on the three months post-operative outcome.

Limitations of our study include a retrospective analysis with a relatively small sample size and short-term follow-up. However, the authors faced difficulties in recruiting adequate number of HIV-positive patients because of common poor compliance with both the therapies and the follow-up arrangements in several patients. To overcome these difficulties in the matching process the authors modified the Charlson Comorbidity Index omitting the fixed six-point score for HIV-positivity. Likewise the present study is the only one in the literature, performed in a single centre by a single homogeneous surgical team, specifically addressing early outcome of femoral neck fractures in HIV-positive patient matched to a HIV-negative control group.

## Conclusions

There was no statistically significant increase in early complications for femoral neck fracture fixation in HIV-positive compared to HIV-negative patients without the necessity to address these patients in dedicated centres. Only one HIV-positive patient with a poor HAART compliance reported a bad outcome. However, patients with poor adherence to antiretroviral therapy and uncontrolled parameters including viremia, CD4 counts and T-helper lymphocytes counts, could be more likely to face early post-operative complications and to support this concept it is recommended in the future similar larger multicentric study be conducted considering the objective difficulties both in enrolling and matching these patients.
